# High Risk Subgroups Sensitive to Air Pollution Levels Following an Emergency Medical Admission

**DOI:** 10.3390/toxics5040027

**Published:** 2017-10-16

**Authors:** Seán Cournane, Richard Conway, Declan Byrne, Deirdre O’Riordan, Seamus Coveney, Bernard Silke

**Affiliations:** 1 Medical Physics Department, St James’s Hospital (SJH), 8 Dublin, Ireland; 2 Department of Internal Medicine, St James’s Hospital (SJH), 8 Dublin, Ireland; drrichardconway@gmail.com (R.C.); declangbyearsne@me.com (D.B.); doriordan@stjames.ie (D.O.); 3 Envo-Geo Environmental Geoinformatics, Cork, Ireland; seamus@envogeo.ie

**Keywords:** emergency medical admissions, high risk cohort, PM_10_, SO_2_, NO*_x_*

## Abstract

For three cohorts (the elderly, socially deprived, and those with chronic disabling disease), the relationship between the concentrations of particulate matter (PM_10_), sulphur dioxide (SO_2_), or oxides of nitrogen (NO*_x_*) at the time of hospital admission and outcomes (30-day in-hospital mortality) were investigated All emergency admissions (90,423 episodes, recorded in 48,035 patients) between 2002 and 2015 were examined. PM_10_, SO_2_, and NO*_x_* daily levels from the hospital catchment area were correlated with the outcomes for the older admission cohort (>70 years), those of lower socio-economic status (SES), and with more disabling disease. Adjusted for acuity and complexity, the level of each pollutant on the day of admission independently predicted the 30-day mortality: for PM_10_–OR 1.11 (95% CI: 1.08, 1.15), SO_2_–1.20 (95% CI: 1.16, 1.24), and NO*_x_*–1.09 (1.06–1.13). For the older admission cohort (≥70 years), as admission day pollution increased (NO*_x_* quintiles) the 30-day mortality was higher in the elderly (14.2% vs. 11.3%: *p* < 0.001). Persons with a lower SES were at increased risk. Persons with more disabling disease also had worse outcomes on days with higher admission particulate matter (PM_10_ quintiles). Levels of pollutants on the day of admission of emergency medical admissions predicted 30-day hospital mortality.

## 1. Introduction

The World Health Organization (WHO) has estimated that particulate matter (PM) air pollution contributes to approximately 800,000 premature deaths each year, ranking it the 13th leading cause of mortality worldwide [1]. This component of air pollution is made up of extremely small particles and liquid droplets containing acids, organic chemicals, metals, and soil or dust particles. Increased particulate matter (PM) leads to increased mortality, particularly from respiratory and cardiovascular disease [1,2,3,4]. Chronic obstructive pulmonary disease (COPD) patients experience worsening symptoms and increased morbidity in the emergency room when exposed to higher PM mass concentration levels [5,6,7]. In Dublin, over 30 years ago, an association between increases in mortality and poor air pollution was examined [8], leading to the enactment of air pollution control legislation, which subsequently led to reduced average black smoke concentrations, approximating 35.6 μg/m^3^ [9]; mortality decreased by 5.7%, while deaths due to respiratory and cardiovascular illness significantly reduced, providing substantial evidence for the legislation [9]. Despite considerably lower current air pollution levels, pollution below international standards may still prove detrimental to health [3,4,10,11].

This study reports on air pollution levels on the day of a hospital admission and the subsequent outcome (30-day hospital mortality) following an emergency medical admission, examining the outcome of three distinct cohorts. The catchment area of the hospital in this study is mainly an inner city, with over half of patients described as deprived and ageing [12]. Long-term exposure for older persons to PM mass concentrations at levels typically experienced by many individuals in the United States is associated with significantly worse cognitive decline [13]. Ecological studies of environmental equity have shown that groups with lower socioeconomic status (SES) are more likely to be exposed to higher air pollution levels than groups of higher SES in some European cities [14]. Both of these observations may be relevant to the catchment area studied in this work. The hospital in this study also has many patients with chronic disabling diseases [15] whose susceptibility to air pollution levels are unclear. In the context of the emergency medical admission, although each component of air pollution may exert specific toxic effects on the respiratory or cardiovascular systems, oxides of nitrogen (NO*_x_*) and PM are potent oxidants, either directly or indirectly [16]. Oxidative stress may be active during emergency medical admissions; thus, its effect on acute disease is of interest [17]. It has previously been shown at the institution studied in this work that respiratory emergency admissions are susceptible to the levels of air pollutants, with worse outcomes when admission day levels are higher; however, in this study it was investigated whether the current levels of air pollutants, as determined by daily levels of particulate matter (PM_10_), sulphur dioxide (SO_2_), or oxides of nitrogen (NO*_x_*), impact upon the 30-day hospital mortality for all acute medical emergency admissions, and three admission cohorts in particular—older persons (≥70 years), persons with lower socio-economic status (SES), and those with chronic disabling disease.

## 2. Materials and Methods

St. James’ Hospital (SJH) is a secondary care hospital serving a catchment area of approximately 270,000 adults. The catchment area is located mainly in the inner city area of Dublin with a largely aging and deprived population emergency medical admissions are admitted from the Emergency Department to an Acute Medical Admission Unit (AMAU). The operation of the AMAU has been previously detailed in the literature [18,19].

### 2.1. Data Collation

A patient database, which had been anonymised, was utilized in the work. Information included details from the patient administration system (PAS), national hospital in-patient enquiry (HIPE) scheme [20,21], patient electronic record (EPR) and other laboratory databases. We employed the International Classification of Diseases, 9th Rev., Clinical Modification (ICD-9-CM) for diagnosis and procedure coding (1990–2005) and ICD-10-CM since 2005. Data included the unique hospital identifier, consultant at time of admission, patient address, date of birth, gender, principal, and up to nine additional primary and secondary diagnoses, respectively. In addition dates of admission and discharge were available. Further parameters included those physiological, haematological, and biochemical data.

### 2.2. Deprivation Indices

The smallest administrative areas reported on by the census are electoral divisions (EDs). In this work, we used a combination of four indicators to describe deprivation. As per SAHRU investigations, these include, type of housing, unemployment, social class, and car ownership [22]. The deprivation scores used in this work have been described previously [23]. The patient address information allowed for locating the electoral division, which was matched with the Deprivation Index. ArcGIS Geographic Information System software was used for this purpose [24].

### 2.3. Air Quality

In this work, PM_10_, NO*_x_*, and SO_2_ data was collated from three stations (Winetavern and Coleraine Street or Rathmines) within the hospital catchment area over the 2002–2015 period [25]. In order that a reliable measure for each pollutant be recorded, a daily average for each was calculated with the data divided into equally spaced quintiles for PM_10_, NO*_x_* and SO_2_, respectively. In the case where data from a collection station were missing, the value from the site typically recording the highest value was selected. If this was unavailable we proceeded in descending order. Annual trend data was then based on the medians/means of the daily data for each pollutant, respectively.

### 2.4. Risk Predictors and Statistical Methods

We investigated in-hospital mortality (within 30-days of admission) as our primary outcome; this is the risk of death within the first 30-days of admission. An Acute Illness Severity Score (AISS) has previously been derived [26], allowing for the prediction of 30-day in-hospital mortality from laboratory data collated in the Emergency Department. The use of acute Illness severity scores features for emergency and critical care patients in the literature [27,28,29]. At the centre studied in this work, an illness severity score has been developed over decades [18,26]. The score was originally based on the APACHE II score [29] but has been adapted to include more predictive terms [26]. As a result, the data includes: potassium (K), sodium (Na), albumin, urea, red cell distribution width (RDW), and white blood cell count (WCC). Their relationship with in-hospital mortality has been derived as a result and described in detail elsewhere [30]. From the derived weighted score, six risk groups (I–VI) of 30-day in-hospital mortality rates have been set, adjusting for chronic disabling disease [15] Charlson Co-Morbidity [31] and IDC9/ICD10 discharge code. Sepsis categorised according to having no request, a negative culture, or a positive culture was included as evaluated. Each predictor was included as part of the outcome risk for each patient which was adjusted in order to estimate of the pollutants impact on inpatient hospital mortality. This was achieved using a multiple variable logistic regression model.

Descriptive statistics were calculated and presented for demographic data, including means/standard deviations (SD), medians/interquartile ranges (IQR). Comparisons between in-hospital mortality and categorical variables were carried out using chi-square tests; Scheffe’s comparison statistic was employed where multiple comparisons were investigated. We used logistic regression to further investigate those significant univariate outcome predictors (*p* < 0.10) of in-hospital mortality. The logistic regression analysis examined the association between in-hospital mortality and those predictors including: AISS, chronic disabling disease [15], co-morbidity [31], sepsis [32], the Deprivation score and the daily PM_10_, SO_2_, and NO*_x_* levels.

Margins statistics were estimated in Stata in order to investigate adjusted predictions within sub-groups, while controlling for other variables. In the logistic multivariable model, univariate estimates were adjusted for, using the described outcome predictors.

Adjusted odds ratios (OR) and 95% confidence intervals (CI) or incident rate ratios (IRR) were calculated for predictor variables that proved significant (*p* < 0.10). All analyses were performed in Stata v.13.1 (Stata Corporation, College Station, TX, USA).

## 3. Results

This study comprised 90,423 episodes, recorded in 48,035 patients who were admitted as emergency medical admissions between 2002 and 2015. These were all considered for analysis. The percentage of males was 48.9%. The median (IQR) length of stay (LOS) was 6.4 (2.7, 14.2) days. The median (IQR) age was 68.2 (48.1, 80.3) years, with the upper 10% boundary at 86.4. The major disease categories (MDC) were respiratory (25.2%), cardiovascular (16.2%), neurological (16.8%), and gastrointestinal (10.0%). Overall the rate of 30-day mortality during 2002–2015 was 5.1% (95% CI: 4.9%, 5.3%); where unique patients were only considered for respiratory patients the 30-day hospital mortality rate was 11.1% (95% CI: 10.7, 11.4). As described in previous work, the mortality rate at the institution has decreased since the opening of the Acute Medical Assessment Unit [18,19].

### 3.1. Admission PM_10_, SO_2_, and NO_x_ Levels and 30-Day Hospital Mortality (Figure 1, Table 1)

**Figure 1:**
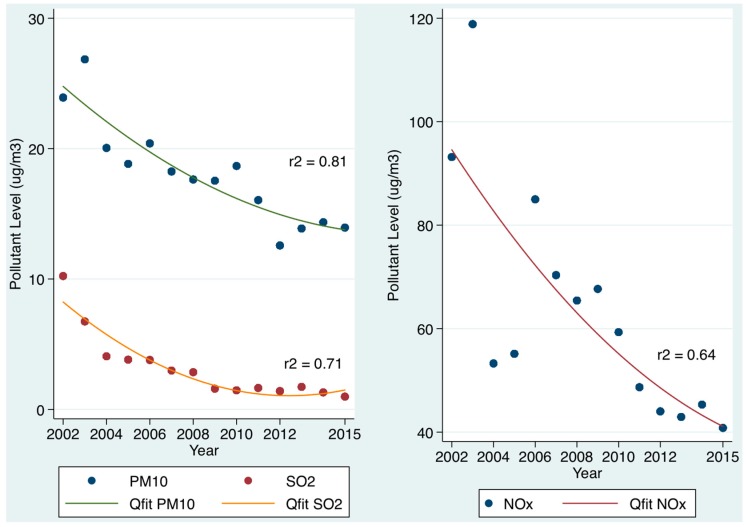
Annual trends of air pollution (PM_10_, NO*_x_*, and SO_2_) over time from 2002 to 2015. For all three pollutants, there annual average levels have declined over time. The trend lines are nonlinear quadratic fits.

There was a significant decrease in pollution levels from 2002 to 2015, as illustrated in Figure 1. The 30-day in-hospital mortality was related to the PM_10_ mass concentration on the admission day to the hospital; we adjusted for other mortality predictors including the acute illness severity, Chronic disabling disease [15], Charlson Co-Morbidity Index [31], Deprivation Index [33], sepsis status [32], and major disease categories (MDC) of respiratory, cardiac and neurological subtypes. A death by day 30 was independently predicted by the PM_10_ mass concentration level on the day of hospital admission–OR 1.11 (95% CI: 1.08, 1.15). Similarly, the SO_2_ level on the day of admission was independently predictive of a 30-day death after adjustment for the acuity and complexity parameters–OR 1.20 (95% CI: 1.16, 1.24). Given the OR was increased for SO_2_ relative to the other pollutants, we examined the changing OR with different quintiles (Table 1). The level of NO*_x_* levels on the day of admission was also independently predictive in the multiple variable model–OR 1.09 (95% CI: 1.06, 1.13).

### 3.2. Air Pollution on Day of Admission and Patient Age (Figure 2)

**Figure 2:**
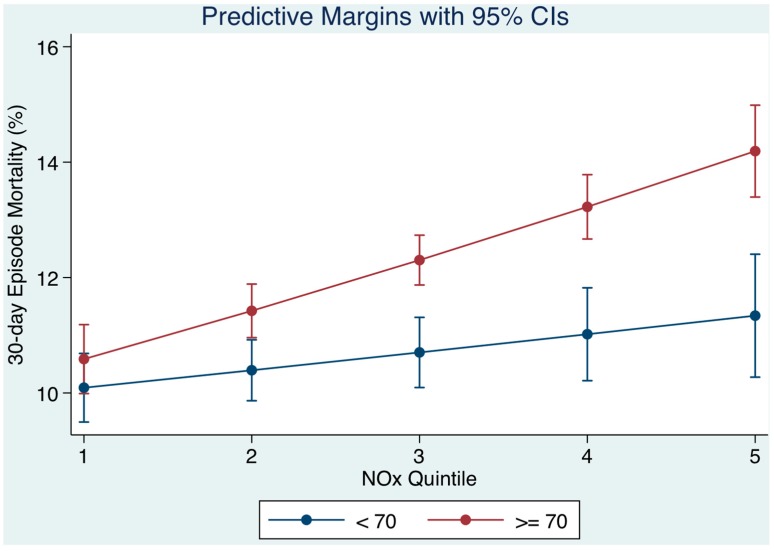
The 30-day in hospital mortality related daily oxides of nitrogen (NO*_x_*) levels by quintiles to the 30-day in-hospital mortality and adjusted for socio-economic status (SES) and other outcome predictors. We used margins to estimate the average marginal effect. As NO*_x_* quintiles increased (level of NO*_x_* on day of admission), the 30-day mortality progressively increase but was higher for older persons.

We calculated the average marginal effect (AME) of the patient’s age, when examining the effect of the level of air pollution on the day of admission and the 30-day mortality outcome. The marginal effect estimates the relationship between unit change in the air pollution at time of presentation and the 30-day mortality outcome—all other variables being held constant. The marginal effect is first calculated for each individual at the observed levels of covariates; these values are then averaged across all individuals to yield the average marginal effect (AME). We divided patients into a younger <70 years and older cohort (≥70 years). As NO*_x_* quintiles increased (level of NO*_x_* on day of admission), the 30-day mortality progressively increased but was higher for older persons. The model prediction for younger patients, 30-day mortality from Q1 to Q5 of NO*_x_* would increase from 10.1% to 11.3% contrasting with older patients whose mortality would increase from 10.6% to 14.2% (Figure 2). The interaction effect between NO*_x_* quintile and age (< or ≥70 years) was 1.07 (95% CI: 1.04, 1.10).

### 3.3. Relation of Socio-Economic Status on Day of Admission (Figure 3)

**Figure 3:**
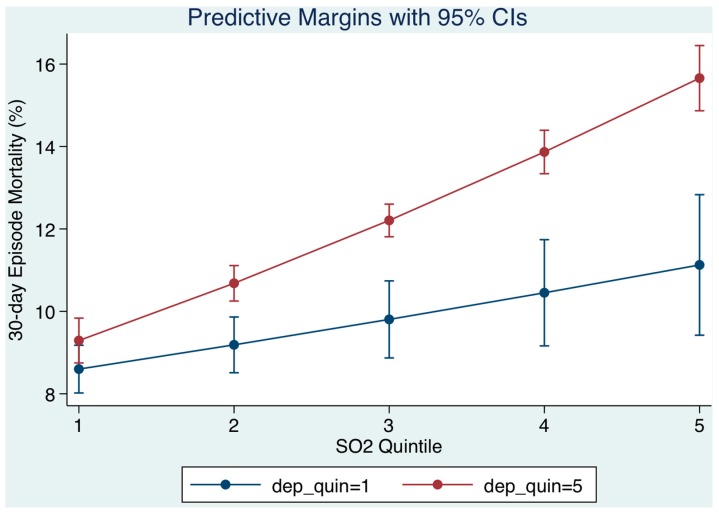
The 30-day in hospital mortality related daily sulphur dioxide (SO_2_) levels by quintiles to the 30-day in-hospital mortality and adjusted for socio-economic status (SES) and other outcome predictors. We used margins to estimate the average marginal effect. As SO_2_ quintiles increased (the level of SO_2_ on day of admission), the 30-day mortality progressively increased, but was higher for patients of lower SES.

We examined the socio-economic status (SES) of patients resident within our catchment area in the multiple variable logistic model and the extent to which this was relevant to the relationship between the level of air pollution at time of presentation and the 30-day hospital mortality outcome. The deprivation score was utilized to categorise residential small areas into quintiles, from lowest (least deprived) to highest (most deprived). As SO_2_ quintiles increased (level on day of admission), the 30-day mortality progressively increased but was higher for patients of lower SES. The model prediction was that for persons from the least deprived areas (Q1 deprivation), 30-day mortality from Q1 to Q5 of SO_2_ would increase from 8.6% to 11.1% contrasting with persons from more deprived areas (Q5 deprivation) whose mortality would increase from 9.3% to 15.7% (Figure 3). The interaction effect between SO_2_ quintile and SES (lower or higher) was 1.03 (95% CI: 1.01, 1.04).

### 3.4. Relation of Disabling Disease Status on Day of Admission (Figure 4)

**Figure 4:**
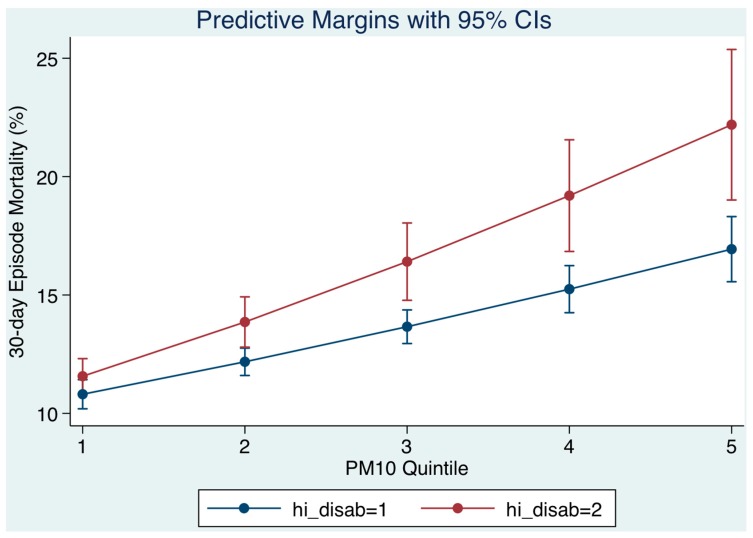
The 30-day in hospital mortality related daily particulate matter (PM_10_) levels by quintiles to the 30-day in-hospital mortality and adjusted for chronic disabling disease and other outcome predictors. Disabling disease was classified with scores of 1, 2, 3, and 4+ based on the number of organ systems involved; the cut point between less or more was >3. At higher PM_10_ mass concentration quintiles (level of PM_10_ on day of admission), the 30-day mortality progressively increased but was higher for patient with more disabling disease.

We examined the Chronic Disabling Disease status of patients resident within our catchment area in the multiple variable logistic model and the extent to which this was relevant to the relationship between the level of air pollution at time of presentation and the 30-day hospital mortality outcome. Disabling disease was classified with scores of 1, 2, 3, and 4+ based on the number of organ systems involved; the cut point between less or more was >3. As PM_10_ quintiles increased (level on day of admission), the 30-day mortality progressively increased but was higher for patients with more disabling disease. The model prediction was that for persons with less disabling disease (scores 1–3), 30-day mortality from Q1 to Q5 of PM_10_ mass concentrations would increase from 10.8% to 16.9% contrasting with persons with more disabling disease (scores > 3) whose mortality would increase from 11.6% to 22.2% (Figure 4). The interaction effect between PM_10_ quintile and disabling disease category (less or more) was 1.10 (95% CI: 1.07, 1.14).

## 4. Discussion

Studies have shown that long-term exposure to air pollution increases mortality [1,2,3,4]; however, there is limited evidence where the air-pollution levels lie below the most recent National Ambient Air Quality Standards. There has been significant evidence of adverse effects related to exposure to PM_2.5_ and ozone at concentrations below national standards [34]. Our work has considered the impact of atmospheric air pollution on emergency medical admission; the data clearly demonstrated that each pollutant, whether PM_10_, SO_2_, or NO*_x_* levels on the day of hospital admission independently predicted the subsequent 30-day hospital mortality. Adjusted for acuity and complexity, the level of each pollutant, divided into quintiles, on the day of admission independently predicted the 30-day mortality. This study did not attempt to determine which of the individual pollutants were more or less problematic; each pollutant was simply regressed against the outcome (30-day in-hospital mortality) after adjustment for other outcome predictors such as the Acute Illness Severity, Charlson Co-Morbidity Index [31], and Chronic Disabling Disease [15], and sepsis status [32].

The catchment area of our centre lies mainly in the inner city with over half the patients described as deprived [12]; 47 of the 74 electoral division areas considered in the highest quintile of the National Deprivation Index [22]. It is important to note that admissions from areas of low SES tend to be considerably younger than admissions from more affluent areas; in this study the median for lower SES (Q3/Q5 SES) was 66.3 years (95% CI: 46.7, 79.5), more than a decade younger than those admitted from higher SES (Q1/Q2 SES), 78.1 years (95% CI: 65.5, 84.8). Nevertheless, most ecological studies of environmental equity show that groups with lower SES are more likely to be exposed to higher air pollution levels than groups of higher SES [14]. A previous cohort study linked improvements in air pollution levels over 11 years to attenuation of the decline in lung function, providing important insight into causality between long-term air pollution and exposure [35]. The ESCAPE project related long-term exposure to ambient air pollution with the level of lung function across different cities and regions in Europe. Impaired lung function exhibited the most consistent association with different pollution metrics being inversely related to nitrogen oxides and PM_10_ mass concentrations, as well as to traffic load at the residential address [36]. Further, significant associations between PM_10_, SO_2_, and NO*_x_* and all non-accidental and cardiovascular mortality have been reported in areas of middle or high socioeconomic deprivation [37]. Our data are in agreement with the literature echoing that neighbourhood socio-economic deprivation increases mortality risks associated with air pollution. It is possible that these poor outcomes were attributable to an increased level of exposure rather than community disadvantage [38]; however, the three monitoring stations provide average geographical exposure estimates. Of course, our data operate over a comparatively short period and might be consistent with the hypothesis that at a time of acute illness deterioration oxidative stress might be important [16]. Oxidative stress may be active during an emergency medical admission; there is interest in the role that oxidative stress may play for acute disease [17].

We investigated two other cohorts—older persons and those with chronic disabling disease. For older persons, long-term exposure to traffic-related air pollution increases the risk for asthma hospitalisation; those with previous asthma or COPD hospitalisations are most susceptible [39]. Exposure to PM has been associated with an accelerated cognitive decline in older women [13]. Further, a review of the main health effects of pollutants in the elderly indicated higher risks compared to the rest of the population. Increased pollution exposures have associated with increased numbers of hospital admissions and emergency-room visits and, indeed, mortality for cardio-pulmonary or respiratory causes. This has been attributed to exacerbations of COPD and asthma, or to respiratory tract infections such as pneumonia, with episodes related to increased incidence of respiratory diseases, and decreased lung function [40]. Our multiple variable model, adjusted for disease acuity, complexity, and comorbidities, predicted that, for younger patients, 30-day mortality from across the pollutant quintiles would increase 30-day hospital mortality from 10.1% to 11.3%, contrasting with older patients whose mortality would increase from 10.6% to 14.2%. Thus, it is suggested that older acute admissions are at an increased risk for a worse outcome on days with pollution levels in the higher quintiles, albeit these levels may be within the current air quality guideline limits.

For the disabling disease cohort, we have previously calibrated a disabling disease score in our emergency medical admissions [15] based on the definition proposed by the US Department of Health and Human Services for a ‘chronic disabling condition’ [41]. Scores of 1, 2, 3, and 4+, classified by the number of organ systems involved, were associated with increasing 30-day mortality rates of 2.6%, 4.1%, 6.3%, and 10.9%; high ‘disability’ and illness severity predicted a particularly bad outcome. In this work we reveal a new finding that patients with disabling disease are more sensitive to air pollution; as the pollutant quintiles increased 30-day hospital mortality for those with less disabling disease was predicted to range from 10.8% to 16.9%, contrasting with more disabling disease whose mortality would increase from 11.6% to 22.2%. Thus, for those with increased levels of disabling disease, with more than three organ systems involved, the 30-day mortality was higher. Once again, this may be due to the influence of oxidative stress affecting patients who are suffering a number of underlying illnesses.

Data of an emergency medical admission cohort has been utilised, consisting of data gathered from a number of years of admissions. The study, however, has been carried out in one hospital, which may limit generalisablity of the findings to the population as a whole. In addition, patients from areas of less deprivation may be present as emergencies in the private health system. As a result we have no way of including these patients in our statistics. Additionally, they may lead to an underrepresentation of the admission incidence rates of patients from the more affluent areas. Furthermore, we have relied on average daily pollutant levels in our catchment area and, thus, did not have access to more detailed data across each of the electoral divisions.

## 5. Conclusions

Over the study period a decrease in PM_10_, SO_2_, and NO*_x_* pollutant levels, within levels regarded internationally as low, were observed in the investigated hospital catchment area. High risk groups (older persons, those of low SES status, or with more debilitating disease) were examined to establish whether they had a worse outcome if admitted to hospital on days with higher levels of air pollution, by quintile. It was found that for the older admission cohort (≥70 years), as admission day pollution increased (NO*_x_* quintiles) the 30-day mortality was higher in the elderly. Furthermore, those in the lower SES groups and those patients with more disabling disease were at increased risk with increased quintiles of pollution on the day of their admission. Thus, it has been shown that there is an adverse effect on at risk groups where there is an increase in levels of pollutants on the day of admission to the hospital.

## Figures and Tables

**Table 1:** Logistic multivariable model of 30-day in hospital mortality related to admission levels of PM_10_ and SO_2_, with adjustment for other risk predictors.

Parameter	Group	OR	95% CI	*p* <
SO_2_ (µg/m^3^)	Q II	1.30	1.12, 1.50	0.001
	Q III	1.54	1.33, 1.78	0.001
	Q IV	1.92	1.66, 2.22	0.001
	Q V	1.88	1.60, 2.21	0.001
PM_10_ (µg/m^3^)		1.04	1.01, 1.08	0.02
Illness Severity		3.97	3.58, 4.41	0.001
Charlson Index		1.56	1.47, 1.66	0.001
Disabling Score		1.19	1.14, 1.25	0.001
Sepsis		2.44	2.28, 2.63	0.001
MDC Respiratory		1.98	1.77, 2.22	0.001
MDC Cardiac		1.72	1.51, 1.96	0.001
MDC Neurology		2.13	1.84, 2.48	0.001
Deprivation—ED		1.09	1.05, 1.14	0.001

## References

[1] Anderson J.O., Thundiyil J.G., Stolbach A. (2011). Clearing the Air: A Review of the Effects of Particulate Matter Air Pollution on Human Health. J. Med. Toxicol..

[2] Samet J.M., Dominici F., Curriero F.C., Coursac I., Zeger S.L. (2000). Fine particulate air pollution and mortality in 20 U.S. cities, 1987–1994. N. Engl. J. Med..

[3] Katsouyanni K., Touloumi G., Spix C., Schwartz J., Balducci F., Medina S., Rossi G., Wojtyniak B., Sunyer J., Bacharova L. (1997). Short-term effects of ambient sulphur dioxide and particulate matter on mortality in 12 European cities: Results from time series data from the APHEA project. Air Pollution and Health: A European Approach. BMJ.

[4] Dockery D.W., Pope C.A. (1994). Acute respiratory effects of particulate air pollution. Annu. Rev. Public Health.

[5] Pope C.A., Kanner R.E. (1993). Acute Effects of PM_10_ Pollution on Pulmonary Function of Smokers with Mild to Moderate Chronic Obstructive Pulmonary Disease. Am. Rev. Respir. Dis..

[6] Atkinson R.W., Anderson H.R., Sunyer J., Ayearses J., Baccini M., Vonk J.M., Boumghar A., Forastiere F., Forsberg B., Touloumi G. (2001). Acute effects of particulate air pollution on respiratory admissions: Results from APHEA 2 project. Air Pollution and Health: A European Approach. Am. J. Respir. Crit. Care Med..

[7] Dominici F., Peng R.D., Bell M.L., Pham L., McDermott A., Zeger S.L., Jonathan M., Samet M.D. (2006). Fine particulate air pollution and hospital admission for cardiovascular and respiratory diseases. JAMA.

[8] Kelly I., Clancy L. (1984). Mortality in a general hospital and urban air pollution. Ir. Med. J..

[9] Clancy L., Goodman P., Sinclair H., Dockery D.W. (2002). Effect of air-pollution control on death rates in Dublin, Ireland: An intervention study. Lancet.

[10] Schwartz J. (1994). Air pollution and daily mortality: a review and meta analysis. Environ. Res..

[11] Katsouyanni K., Zmirou D., Spix C., Sunyer J., Schouten J.P., Ponka A., Anderson H.R., Le Moullec Y., Wojtyniak B., Vigotti M.A. (1995). Short-term effects of air pollution on health: A European approach using epidemiological time-series data. The APHEA project: background, objectives, design. Eur. Respir. J..

[12] Walsh J.B., Coakley D., Murphy C., Coakley J.D., Boyle E., Johnson H. (2004). Demographic profile of the elderly population in Dublin accident and emergency hospital catchment areas. Ir. Med. J..

[13] Weuve J., Puett R.C., Schwartz J., Yanosky J.D., Laden F., Grodstein F. (2012). Exposure to particulate air pollution and cognitive decline in older women. Arch. Intern. Med..

[14] Havard S., Deguen S., Zmirou-Navier D., Schillinger C., Bard D. (2009). Traffic-Related Air Pollution and Socioeconomic Status. Epidemiology.

[15] Chotirmall S.H., Picardo S., Lyons J., D’Alton M., O’Riordan D., Silke B. (2014). Disabling disease codes predict worse outcomes for acute medical admissions. Intern. Med. J..

[16] Lodovici M., Bigagli E. (2011). Oxidative stress and air pollution exposure. J. Toxicol..

[17] Bar-Or D., Bar-Or R., Rael L.T., Brody E.N. (2015). Oxidative stress in severe acute illness. Redox Biol..

[18] Rooney T., Moloney E.D., Bennett K., O’Riordan D., Silke B. (2008). Impact of an acute medical admission unit on hospital mortality: A 5-year prospective study. QJM.

[19] Conway R., O’Riordan D., Silke B. (2014). Long-term outcome of an AMAU—A decade’s experience. Q. J. Med..

[20] O’Loughlin R., Allwright S., Barry J., Kelly A., Teljeur C. (2005). Using HIPE data as a research and planning tool: Limitations and opportunities. Ir. J. Med. Sci..

[21] O’Callaghan A., Colgan M.P., McGuigan C., Smyth F., Haider N., O’Neill S., Moore D., Madhavan P. (2012). A critical evaluation of HIPE data. Ir. Med. J..

[22] Kelly A., Teljeur C. (2007). SAHRU National Deprivation Index Trinity College, Dublin,. http://www.sahru.tcd.ie/services/deprivation/DeprivationFiles/WebReport07.pdf.

[23] Conway R., Galvin S., Coveney S., O’Riordan D., Silke B. (2013). Deprivation as an outcome determinant in emergency medical admissions. QJM.

[24] Shimrat M. (1962). Algorithm 112: Position of point relative to polygon. Commun. ACM.

[25] Keary J., Jennings S.G., O’Connor T.C., McManus B., Lee M. (1998). PM_10_ concentration measurements in Dublin city. Environ. Monit. Assess..

[26] Silke B., Kellett J., Rooney T., Bennett K., O’Riordan D. (2010). An improved medical admissions risk system using multivariable fractional polynomial logistic regression modelling. Q. J. Med..

[27] Vincent J.L., Moreno R. (2010). Clinical review: Scoring systems in the critically ill. Crit. Care.

[28] Williams J.M., Greenslade J.H., Chu K., Brown A.F., Lipman J. (2016). Severity Scores in Emergency Department Patients With Presumed Infection: A Prospective Validation Study. Crit. Care Med..

[29] Knaus W.A., Draper E.A., Wagner D.P., Zimmerman J.E. (1985). APACHE II: A severity of disease classification system. Crit. Care Med..

[30] Courtney D., Conway R., Kavanagh J., O’Riordan D., Silke B. (2014). High-sensitivity troponin as an outcome predictor in acute medical admissions. Postgrad. Med. J..

[31] Charlson M.E., Pompei P., Ales K.L., MacKenzie C.R. (1987). A new method of classifying prognostic comorbidity in longitudinal studies: Development and validation. J. Chronic Dis..

[32] Chotirmall S.H., Callaly E., Lyons J., O’Connell B., Kelleher M., Byearsne D., O’Riordan D., Silke B. (2016). Blood cultures in emergency medical admissions: A key patient cohort. Eur. J. Emerg. Med..

[33] Kelly A., Teljeur C. (2007). SAHRU National Deprivation Index.

[34] Di Q., Wang Y., Zanobetti A., Wang Y., Koutrakis P., Choirat C., Dominici F., Schwartz J.D. (2017). Air Pollution and Mortality in the Medicare Population. N. Engl. J. Med..

[35] Downs S.H., Schindler C., Liu L.J., Keidel D., Bayer-Oglesby L., Brutsche M.H., Gerbase M.W., Keller R., Künzli N., Leuenberger P. (2007). Reduced exposure to PM_10_ and attenuated age-related decline in lung function. N. Engl. J. Med..

[36] Adam M., Schikowski T., Carsin A.E., Cai Y., Jacquemin B., Sanchez M. (2015). Adult lung function and long-term air pollution exposure. ESCAPE: A multicentre cohort study and meta-analysis. Eur. Respir. J..

[37] Wong C.-M., Ou C.-Q., Chan K.-P., Chau Y.-K., Thach T.-Q., Yang L., Chung R.Y.-N., Thomas G.N., Peiris J.S.M., Wong T.-W. (2008). The Effects of Air Pollution on Mortality in Socially Deprived Urban Areas in Hong Kong, China. Environ. Health Perspect..

[38] Sloggett A., Joshi H. (1994). Higher mortality in deprived areas: Community or personal disadvantage?. BMJ.

[39] Andersen Z.J., B nnelykke K., Hvidberg M., Jensen S.S., Ketzel M., Loft S., Mette S., Anne T., Kim O., Ole R.-N. (2011). Long-term exposure to air pollution and asthma hospitalisations in older adults: A cohort study. Thorax.

[40] Simoni M., Baldacci S., Maio S., Cerrai S., Sarno G., Viegi G. (2015). Adverse effects of outdoor pollution in the elderly. J. Thorac Dis..

[41] Ozminkowski R.J., Smith M.W., Coffey R.M., Mark T.L., Neslusan C.A., Drabek J. (2000). Private Payers Serving Individuals with Disabilities and Chronic Conditions.

